# Elevated vascular transformation blood biomarkers in Long-COVID indicate angiogenesis as a key pathophysiological mechanism

**DOI:** 10.1186/s10020-022-00548-8

**Published:** 2022-10-10

**Authors:** Maitray A. Patel, Michael J. Knauer, Michael Nicholson, Mark Daley, Logan R. Van Nynatten, Claudio Martin, Eric K. Patterson, Gediminas Cepinskas, Shannon L. Seney, Verena Dobretzberger, Markus Miholits, Brian Webb, Douglas D. Fraser

**Affiliations:** 1 Epidemiology and Biostatistics, Western University, London, ON N6A 3K7 Canada; 2 Pathology and Laboratory Medicine, Western University, London, ON N6A 3K7 Canada; 3 Medicine, Western University, London, ON N6A 3K7 Canada; 4 Computer Science, Western University, London, ON N6A 3K7 Canada; 5 Lawson Health Research Institute, London, ON N6C 2R5 Canada; 6 Medical Biophysics, Western University, London, ON N6A 3K7 Canada; 7 Thermo Fisher Scientific, Vienna, Austria; 8 Thermo Fisher Scientific, Rockford, IL USA; 9 Pediatrics, Western University, London, ON N6A 3K7 Canada; 10 Clinical Neurological Sciences, Western University, London, ON N6A 3K7 Canada; 11 Physiology and Pharmacology, Western University, London, ON N6A 3K7 Canada; 12 London Health Sciences Centre, Room C2–C82, 800 Commissioners Road East, London, ON N6A 5W9 Canada

**Keywords:** Long-COVID, Vascular transformation, Angiogenesis, Biomarkers, Machine learning

## Abstract

**Background:**

Long-COVID is characterized by prolonged, diffuse symptoms months after acute COVID-19. Accurate diagnosis and targeted therapies for Long-COVID are lacking. We investigated vascular transformation biomarkers in Long-COVID patients.

**Methods:**

A case–control study utilizing Long-COVID patients, one to six months (median 98.5 days) post-infection, with multiplex immunoassay measurement of sixteen blood biomarkers of vascular transformation, including ANG-1, P-SEL, MMP-1, VE-Cad, Syn-1, Endoglin, PECAM-1, VEGF-A, ICAM-1, VLA-4, E-SEL, thrombomodulin, VEGF-R2, VEGF-R3, VCAM-1 and VEGF-D.

**Results:**

Fourteen vasculature transformation blood biomarkers were significantly elevated in Long-COVID outpatients, versus acutely ill COVID-19 inpatients and healthy controls subjects (P < 0.05). A unique two biomarker profile consisting of ANG-1/P-SEL was developed with machine learning, providing a classification accuracy for Long-COVID status of 96%. Individually, ANG-1 and P-SEL had excellent sensitivity and specificity for Long-COVID status (AUC = 1.00, P < 0.0001; validated in a secondary cohort). Specific to Long-COVID, ANG-1 levels were associated with female sex and a lack of disease interventions at follow-up (P < 0.05).

**Conclusions:**

Long-COVID patients suffer prolonged, diffuse symptoms and poorer health. Vascular transformation blood biomarkers were significantly elevated in Long-COVID, with angiogenesis markers (ANG-1/P-SEL) providing classification accuracy of 96%. Vascular transformation blood biomarkers hold potential for diagnostics, and modulators of angiogenesis may have therapeutic efficacy.

**Supplementary Information:**

The online version contains supplementary material available at 10.1186/s10020-022-00548-8.

## Introduction

Coronavirus disease 2019 (COVID-19) is caused by the highly transmissible severe acute respiratory syndrome coronavirus 2 (SARS-CoV-2) (Harrison et al. 2020). SARS-CoV-2 binds the cell surface angiotensin-converting enzyme 2 (ACE2) receptor, resulting in cell entry and viral replication (Yuki et al. 2020). An innate immune response follows SARS-CoV-2 infection (Koyama et al. 2008) and includes increased interferons, tumor necrosis factor, bradykinin, serine proteases, soluble thrombomodulin and clot lysis times (Garvin et al. 2020; Fraser et al. 2020a; Gill et al. 2020; Juneja 2021; Cani et al. 2019), all contributing to microvascular and thrombotic disease (Fraser et al. 2020b; Ackermann 2020). COVID-19 induces a wide range of disease severity, with hospitalized patients suffering an overall mortality rate of approximately 27% (Bertsimas et al. 2020).

Survivors of COVID-19 often suffer diffuse symptoms that can persist for 2–7 months, referred to as “Long-COVID” (Raveendran et al. 2021; Crook et al. 2021; Davis et al. 2021; Huang et al. 2021). Several mechanisms have been proposed to explain the diffuse symptoms associated with Long-COVID, including the organ-specific expression of ACE-2 receptors that may predispose particular systems to greater tissue injury and prolonged healing (e.g., lung, heart, brain) (Salamanna et al. 2020), and/or microvascular endothelial dysfunction secondary to exacerbated inflammation and thrombotic mechanisms (Fraser et al. 2020b; Gavriilaki et al. 2021). A lack of pathophysiological mechanisms and specific diagnostic markers has led some to question whether Long-COVID is a true disease entity (Matta et al. 2022). Given the ongoing debate associated with Long-COVID diagnoses, and the fact that only symptomatic treatments are available, two major priorities to optimize care of Long-COVID patients include early disease recognition with specific diagnostics, as well as, identification of molecular mechanisms for future targeted therapies.

The overall aim of the study is to determine whether vasculature transformation is active in Long-COVID individuals  one to six months post-infection. Our specific objectives were: 1) to measure a large number of vascular transformation blood biomarkers from patients with Long-COVID, as compared to acutely ill COVID-19 patients and healthy control subjects; 2) to determine the relative importance of specific vascular transformation biomarkers to predict Long-COVID; and 3) to determine any relationships between vascular transformation blood biomarkers and Long-COVID demographic and clinical factors. The vascular transformation biomarkers investigated were chosen to provide a functional survey of angiogenesis, endothelial and platelet activation, coagulation and vascular junctional integrity and permeability (Additional file 1: Table S1).

## Methods

### Study participants and blood sampling

All patients were screened and enrolled from our tertiary care system, The London Health Sciences Centre (London, Ontario, Canada). All patients, both Long-COVID and acutely ill COVID-19, had their COVID-19 status confirmed as part of standard hospital testing by detection of two SARS-CoV-2 viral genes using polymerase chain reaction (CDC 2019-Novel Coronavirus 2019). Long-COVID outpatients had been referred to a specialty clinic based on prolonged, diffuse symptoms. Venous blood work was drawn once as part of a larger clinical screen, and excess plasma collected for later research analysis by Pathology and Laboratory Medicine (PaLM). Both Ward and intensive care unit (ICU) patients were enrolled on admission to hospital. Blood sampling for inpatients began on admission, Ward or ICU day 1 and repeated on day 3. Daily blood was obtained from critically ill ICU patients via indwelling catheters and if a venipuncture was required, research blood draws were coordinated with a clinically indicated blood draw. In keeping with accepted research phlebotomy protocols for adult patients, blood draws did not exceed maximal volumes (Hrpp 2009). Blood was centrifuged and plasma isolated, aliquoted at 250 µL, and frozen at − 80 °C. All samples remained frozen until use and freeze/thaw cycles were avoided. The healthy control subjects were individuals without disease, acute illness, or prescription medications, and that were previously banked in the Translational Research Centre, London, ON (Directed by Dr. D.D. Fraser; https://translationalresearchcentre.com/) (Brisson et al. 2012; Gillio-Meina et al. 2013).

### Patient demographics, clinical data and cohort matching

Baseline characteristics for Long-COVID, Ward and, ICU patients were recorded and included age, sex, comorbidities, presenting symptoms, interventions, and laboratory measurements. For Long-COVID patients, we recorded both initial infection variables and clinical variables at follow-up clinic visit. For the latter, we focused on lingering symptoms, laboratory values and interventions. For ICU patients, we included standard illness severity scores, including Multiple Organ Dysfunction Score (MODS) (Priestap et al. 2020) and Sequential Organ Failure Assessment scores (Singer et al. 2016). The PaO_2_ to FiO_2_ ratio and chest radiograph findings were recorded for all ICU patients. We also recorded clinical interventions received during the observation period including the use of antibiotics, antiviral agents, systemic corticosteroids, vasoactive medications, venous thromboembolism prophylaxis, antiplatelet, or anticoagulation treatment, renal replacement therapy, high flow oxygen therapy, and mechanical ventilation (invasive and non-invasive). Final participant groups were constructed by age- and sex-matching Long-COVID patients with Ward COVID-19 patients, ICU COVID-19 patients and healthy control subjects.

### Multiplex immunoassay

Concentrations of vascular transformation blood biomarkers were determined in human plasma using two distinct custom multiplexed immunoassay kits according to manufacturer’s instructions (Thermo Fisher Scientific): (1) Endothelial Injury Marker 12-Plex Human ProcartaPlex™ Panel, EPX120-15849-901, (12 target proteins with no pre-dilution; VEGF-A, VEGF-D, VEGF-R2, VEGF-R3, P-SEL, E-Selectin, PECAM-1, ANG-1, MMP-1, Thrombomodulin, Syndecan-1 and VLA-4); and (2) Mix&Match 4plex ProcartaPlex^TM^ Panel (4 target proteins with 1:100 pre-dilution; Endoglin, sICAM-1, sVCAM-1 and VE-Cadherin). Both kits utilized Luminex^®^ xMAP™ fluorescent bead-based technology (Luminex Corp., 12212 Technology Blvd, Austin, TX, 78727, USA). The assay plate was treated according to the manufacturer’s instructions and quantified on a compatible Luminex® system (Bio-Plex™ 200 system, Bio-Rad Laboratories, 1000 Alfred Nobel Drive, Hercules, CA, 94547, USA). All study cohorts were equally distributed within and across immunoassay kits. The upper and lower limit of quantification, the average inter-assay %CV, and the average intra-assay %CV for each biomarker are stated in the manufacturer's Certificate of Analysis (Additional file 1: Table S2).

### Conventional statistics

Patient baseline clinical characteristics were reported as median (IQRs) for continuous variables and frequency (%) for categorical variables. Ward and ICU blood draws from day 1 and day 3 were combined for both groups separately. The individual biomarkers were pairwise compared between healthy controls, Ward patients, ICU Patients, and Long-COVID patients using a Mann–Whitney U test. A Bonferroni correction was applied to avoid multiple comparison complications, with corrected P-values < 0.01 considered to be statistically significant. Individual biomarker boxplots were generated to illustrate the concentration distribution and significance comparison of the biomarker between cohorts.

### Machine learning

For machine learning, a Random Forest classifier was used to classify cohorts by their changes in vascular transformation biomarkers. A Random Forest is a set of decision trees and, consequently, we were able to interrogate this collection of trees to identify the features that have the highest predictive value (viz., those features that frequently appear near the top of the decision tree). To reduce overfitting and maintain a conservative model, three-fold cross-validation with a Random Forest of 10 trees and a maximum depth of three was used (Tang et al. 2018). A Boruta feature reduction algorithm was used to identify the biomarkers with the greatest importance (Kursa and Rudnicki 2010). The common important biomarkers when comparing healthy controls with Long-COVID patients and COVID-19 patients with Long-COVID patients were used to develop a selected biomarker profile.

Receiver operating characteristic (ROC) curves were conducted on the classification results to determine the sensitivity and specificity of individual molecules for predicting Long-COVID status in comparison to healthy controls and COVID-19 patients. Area-under-the-curve (AUC) was calculated as an aggregate measure of protein performance across all possible classification thresholds (Bradley 1997). The biomarker data was visualized with a nonlinear dimensionality reduction on the full data matrix using the t-distributed stochastic nearest neighbor embedding (t-SNE) algorithm. t-SNE assumes that the ‘optimal’ representation of the data lies on a manifold with complex geometry, but a low dimension, embedded in the full-dimensional space of the raw data (Maaten and Hinton 2008).

A pairwise comparison, using the Euclidian distance, was conducted to determine the similarity between subjects across the selected biomarkers (Jambu and Jambu 1991). As such, subjects similar across their selected biomarker profile have a lower Euclidian distance compared to subjects with differing biomarker profiles. The distances were visualized using a heatmap of which the scale was adjusted to counter the extremely large distance outliers which reduce the visibility of the other comparisons. Exploratory analysis was also conducted to determine relationships of the selected biomarkers to Long-COVID clinical measures using a Mann–Whitney U test with patients missing clinical data excluded from the analysis. All analysis was conducted using Python version 3.9.7 and Scikit-Learn version 1.0.1.

### Validation cohort

The validation cohort consisted of consecutive Long-COVID outpatients that had been referred to a specialty clinic based on prolonged, diffuse symptoms, and with venous blood work drawn once as part of a larger clinical screen. There was no selection process. Healthy controls were used for comparison and were subsequently matched for age and sex proportion.

### Validation immunoassay

Singleplex enzyme-linked Immunosorbent Assays (ELISAs; Thermo Fisher Scientific) were used to validate the multiplex ELISA results. Plasma samples were thawed on ice and diluted in the manufacturer’s provided dilutant at 1:10 for human ANG-1 (# EHANGPT1) and at 1:40 for soluble human P-SEL (# BMS219-4). All processing was according to the manufacturer’s protocol. Only one Long-COVID P-SEL sample had an optical density (OD) value above the range of the plate reader and was assigned the highest measurable OD. Of the healthy control subject samples, 4 had ANG-1 OD values that fell well below the lowest standard (assigned a 0).

## Results

A total of 4 age- and sex-matched groups were included consisting of Long-COVID outpatients (median years old = 61; IQR = 19; n = 23), Ward COVID-19 inpatients (median years old = 60; IQR = 20; n = 23), ICU COVID-19 inpatients (median years old = 60; IQR = 17; n = 23) and health control subjects (median years old = 59; IQR = 16; n = 23). There were no significant differences with regards to age (P = 0.9869) and sex (P = 1.0000) between the 4 cohorts. Baseline demographic characteristics, comorbidities, laboratory measurements, interventions, and chest x-ray findings of Long-COVID outpatients and the Ward/ICU COVID-19 inpatients, are reported in Tables 1 and 2, respectively. Long-COVID outpatients had a single blood draw at their clinic visit, whereas blood from Ward and ICU COVID-19 inpatients was drawn on day 1 and day 3. Long-COVID patients had significantly elevated lymphocyte measurements in comparison to both Ward and ICU COVID-19 patients as determined by a Kruskal–Wallis H-test (p < 0.0001). The mortality rates for Ward and ICU COVID-19 inpatients were 8.7% and 47.8%, respectively.

**Table 1:** Long COVID-19 outpatient demographics and clinical data

Initial infection variable	Outpatients (n = 23)
Age (yrs), median (IQR)	61.0 (19.0)
Male sex, no. (%)	13 (56.5)
Diagnostic test: PCR, serology, no. (%)	23 (100.0)
Vaccination status at infection, no. (%)	2 (8.7)
Hospitalization, no. (%)	
Ward	7 (30.4)
ICU	1 (4.3)
Comorbidities, no. (%)	
Diabetes	6 (26.1)
Hypertension	8 (34.8)
Coronary artery/heart disease	2 (8.7)
Chronic/congestive heart failure	0 (0.0)
Chronic kidney disease	0 (0.0)
Cancer	1 (4.3)
COPD	0 (0.0)
Asthma	4 (17.4)
Presenting symptoms at infection, no. (%)	
Fever	16 (69.6)
Cough	18 (78.3)
Anosmia/ageusia	14 (60.9)
Pharyngitis	9 (39.1)
Headache	14 (60.9)
Confusion/memory	2 (8.7)
Myalgias	13 (56.5)
Dyspnea	16 (69.6)
Chest pain	8 (34.8)
Nausea/vomiting/diarrhea	12 (52.2)
Interventions at infection, no. (%)	
Steroids	7 (30.4)
Remdesivir	1 (4.3)
Tocilizumab	1 (4.3)
Long-COVID clinic variables	
Follow up, days from infection onset, median (IQR)	98.5 (47.5)
Lingering symptoms at follow up, no. (%)	
Respiratory	16 (69.6)
Cardiovascular	6 (26.1)
Neurology	9 (39.1)
Musculoskeletal	1 (4.3)
Gastro-Intestinal	3 (13.0)
Psychiatric	1 (4.3)
Cutaneous	0 (0.0)
Balance	0 (0.0)
Chest pain	4 (17.4)
Concentration	1 (4.3)
Cough	2 (8.7)
Dyspnea	16 (69.6)
Fatigue	11 (47.8)
Headache	2 (8.7)
Low mood	1 (4.3)
Anxiety	1 (4.3)
Memory	7 (30.4)
Nausea	1 (4.3)
Palpitations	1 (4.3)
Paresthesia	1 (4.3)
Smell/taste	2 (8.7)
Word finding	2 (8.7)
Non-specific	11 (47.8)
Laboratories at follow up, median (IQR)	
White blood cell count	7.1 (2.0)
Neutrophils	4.5 (1.6)
Lymphocytes	2.0 (0.6)
Hemoglobin	140.0 (22.5)
Platelets	230.0 (59.5)
C-Reactive Protein (CRP)	1.8 (3.5)
Ferritin	86.5 (133.8)
Lactate dehydrogenase (LDH)	201.0 (37.0)
Alanine aminotransferase (ALT)	20.0 (10.5)
Interventions at follow up, no. (%)	
Pulmicort	1 (4.3)
Anticoagulant	1 (4.3)
Symbicort	10 (43.5)
Ventolin	3 (13.0)
Lasix	1 (4.3)
Nasal spray	2 (8.7)
Oxygen	2 (8.7)
Physiotherapy	5 (21.7)
None	8 (34.8)

**Table 2:** Acutely ill COVID-19 inpatient demographics and clinical data

Variable	Ward inpatients (n = 23)	ICU inpatients (n = 23)
Age (yrs), median (IQR)	60.0 (20.0)	60.0 (17.0)
Male sex, no. (%)	13 (56.5)	13 (56.5)
Weight (kg), median (IQR)	86.0 (13.4)	89.8 (26.5)
Height (cm), median (IQR)	170.0 (8.0)	171.0 (8.5)
BMI, median (IQR)	28.1 (5.4)	30.3 (7.1)
MODS, median (IQR)	–	5.0 (1.8)
SOFA score, median (IQR)	–	6.0 (5.5)
Comorbidities, no. (%)		
Diabetes	4 (17.4)	10 (43.5)
Hypertension	9 (39.1)	10 (43.5)
Coronary artery/heart disease	1 (4.3)	2 (8.7)
Chronic/congestive heart failure	0 (0.0)	0 (0.0)
Chronic kidney disease	1 (4.3)	2 (8.7)
Cancer	3 (13.0)	2 (8.7)
COPD	0 (0.0)	1 (4.3)
Presenting symptoms, no. (%)		
Fever	18 (78.3)	–
Cough	19 (82.6)	–
Anosmia/ageusia	5 (21.7)	–
Pharyngitis	5 (21.7)	–
Headache	3 (13.0)	–
Myalgias	14 (60.9)	–
Dyspnea	20 (87.0)	–
Chest pain	3 (13.0)	–
Nausea/vomiting/diarrhea	10 (43.5)	–
Pulmonary pathology, no. (%)		
Unilateral pneumonia	–	1 (4.3)
Bilateral pneumonia	22 (95.7)	21 (91.3)
Interstitial infiltrates/R effusion	–	1 (4.3)
Laboratories, median (IQR)		
Hemoglobin	130.0 (24.0)	119.0 (28.5)
White blood cell count	7.0 (5.0)	8.8 (7.5)
Neutrophils	5.9 (4.2)	7.6 (6.9)
Lymphocytes	0.8 (0.6)	0.7 (0.6)
Platelets	219.0 (80.5)	216.0 (131.0)
Creatinine	70.0 (28.5)	81.0 (113.5)
International normalized ratio	1.1 (0.1)	1.2 (0.1)
Lactate	1.7 (1.0)	1.3 (0.8)
Partial thromboplastin time (PTT)	–	27.0 (5.0)
PaO_2_/FiO_2_ Ratio	–	128.0 (70.0)
Interventions, no. (%)		
Renal replacement therapy	0 (0.0)	6 (26.1)
High-flow nasal cannula	13 (56.5)	15 (65.2)
Non-invasive mechanical ventilation	1 (4.3)	7 (30.4)
Invasive mechanical ventilation	2 (8.7)	21 (91.3)
Extracorporeal membrane oxygenation	0 (0.0)	1 (4.3)
Tocilizumab	2 (8.7)	0 (0.0)
Steroids	22 (95.7)	15 (65.2)
Vasoactive medications	2 (9.5)	19 (82.6)
Antibiotics	22 (95.7)	23 (100.0)
Anti-virals	5 (21.7)	3 (13.0)
Antiplatelet	4 (17.4)	18 (78.3)
Anticoagulation	23 (100.0)	22 (95.6)
Outcomes		
Days, median (IQR)	8.0 (7.0)	15.0 (14.0)
Died, no. (%)	2 (8.7)	11 (47.8)

**Fig. 1:**
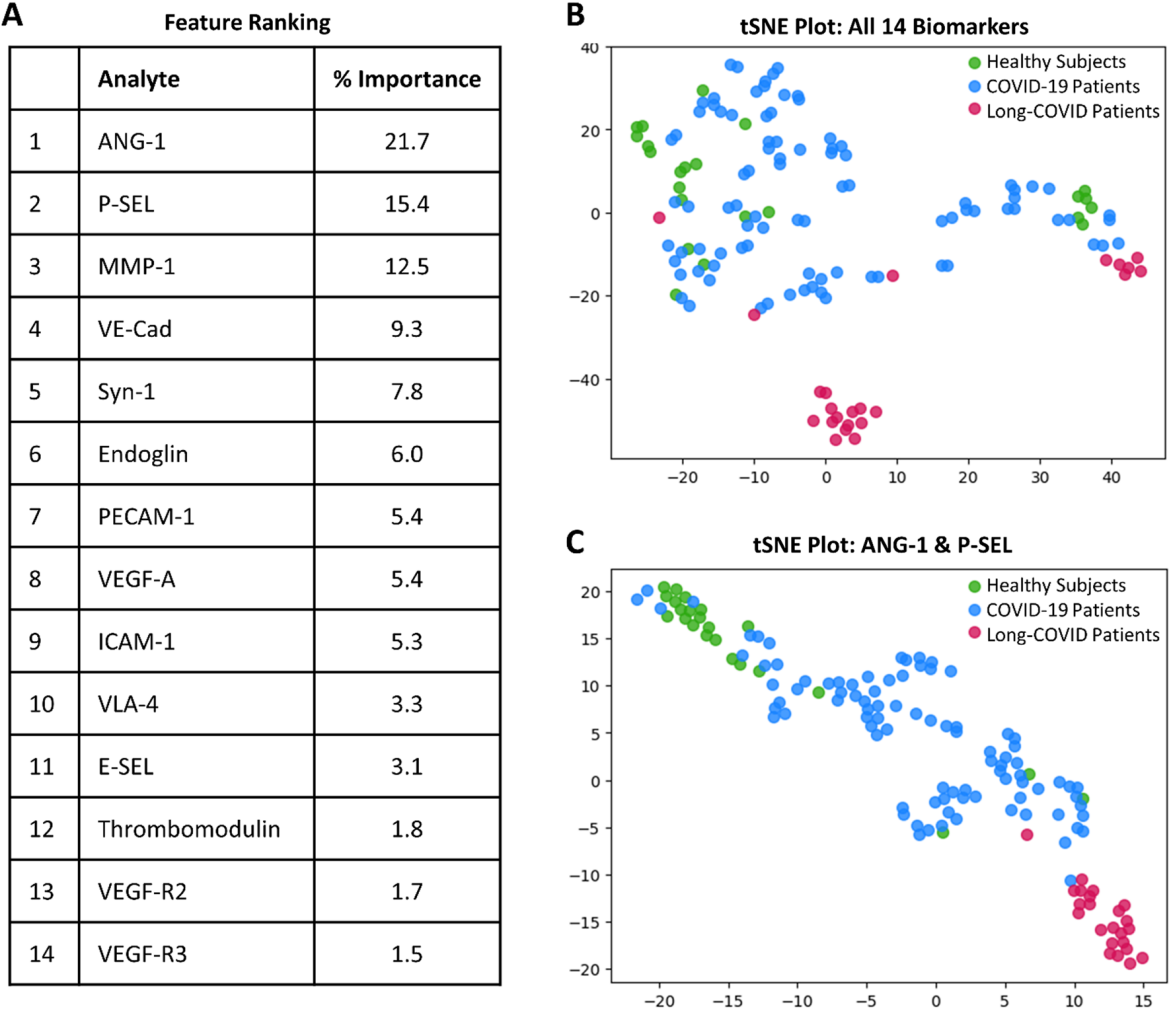
Identification of important vascular transformation blood biomarkers in Long-COVID outpatients. **A** List generated with a Random Forest indicating the relative importance of fourteen blood biomarkers for classifying subjects between cohorts. The leading two biomarkers were ANG-1 and P-SEL. **B** Subjects plotted in two-dimensions, following t-SNE dimensionality reduction of all fourteen significant biomarkers, shows separation cluster of Long-COVID outpatients with some mixing with acutely ill COVID-19 inpatients and healthy control subjects. **C** Subjects plotted in two-dimensions, following t-SNE dimensionality reduction of two selected biomarkers, ANG-1 and P-SEL, showed distinct separation and clustering of Long-COVID outpatients from acutely ill COVID-19 inpatients and healthy control subjects

Sixteen vascular transformation blood biomarkers were measured using multiplex immunoassay technology (Additional file 1: Tables S2 and S3), with fourteen being significantly different between cohorts (P < 0.01 to P < 0.0001; Fig. 1A; Additional file 1: Table S4). Using all 14 vascular transformation blood biomarkers, a t-SNE plot illustrated that Long-COVID patients were easily separable from acutely ill COVID-19 inpatients and healthy control subjects (Fig. 1A, B; classification accuracy 88%). A shortlisted biomarker model was created with Boruta feature reduction and included ANG-1 and P-SEL. The selected model using only ANG-1 and P-SEL produced a t-SNE plot illustrating a separation more distinct between the three cohort groups, with a near-perfect separation of Long-COVID patients (Fig. 1C; classification accuracy 96%). The addition of MMP-1 to the combination of ANG-1 and P-SEL only increased the classification accuracy to 98%.

**Fig. 2:**
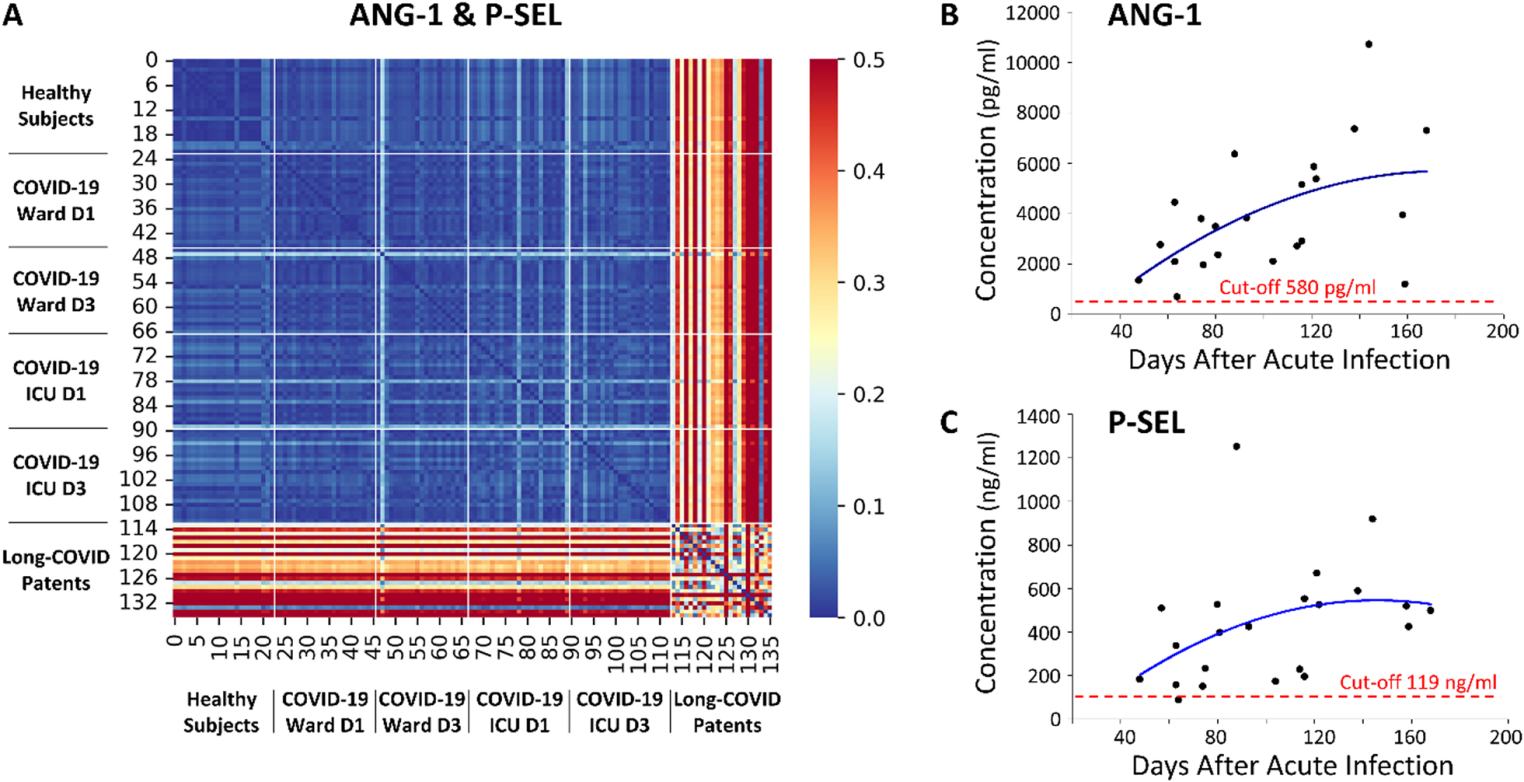
Similar biomarker profiles and plasma concentrations relative to days after acute infection. **A** A heatmap demonstrated the pairwise Euclidian Distance between cohort’s biomarker profiles with respect to ANG-1 and P-SEL. Lower distances between patients indicate similar biomarker profiles while larger distances indicate large differences between profiles (distance was pseudocolored on the bar scale). The biomarker profile of Long-COVID outpatients is distinctively different from all other cohorts. The heatmap color scale was capped at 0.5 to restrict interpretation bias from Long-COVID outliers (max value 1.2) and allow for more visible details. **B** A plot demonstrated ANG-1 concentration versus time after acute infection. A cut-off value, adopted from previous ROC analyses on multiplex data, and a best fit polynomial regression line were calculated. **C** A plot demonstrated P-SEL concentration versus time after acute infection. A cut-off value, adopted from previous ROC analyses on multiplex data, and a best fit polynomial regression line were calculated

To compare the cohorts in terms of a holistic profile containing the selected 2 biomarkers, ANG-1 and P-SEL, Euclidean distances between all subjects were calculated and demonstrated distinct biomarker profiles represented by larger distances between subjects (Fig. 2A). The biomarker profiles of healthy control subjects, Ward COVID-19 inpatients and ICU COVID-19 inpatients were homogeneous as shown by the low Euclidean distances. Although the biomarker profile of all Long-COVID patients is not homogenous, there was a distinct difference between the biomarker profile of Long-COVID patients and that of the other patient cohorts. The plasma concentrations of the two biomarkers in Long-COVID patients, ANG-1 (Fig. 2B) and P-SEL (Fig. 2C), relative to the time after acute infection, were plotted. Cut-off values calculated from the ROC analyses (Additional file 1: Tale S5) were added.

**Fig. 3:**
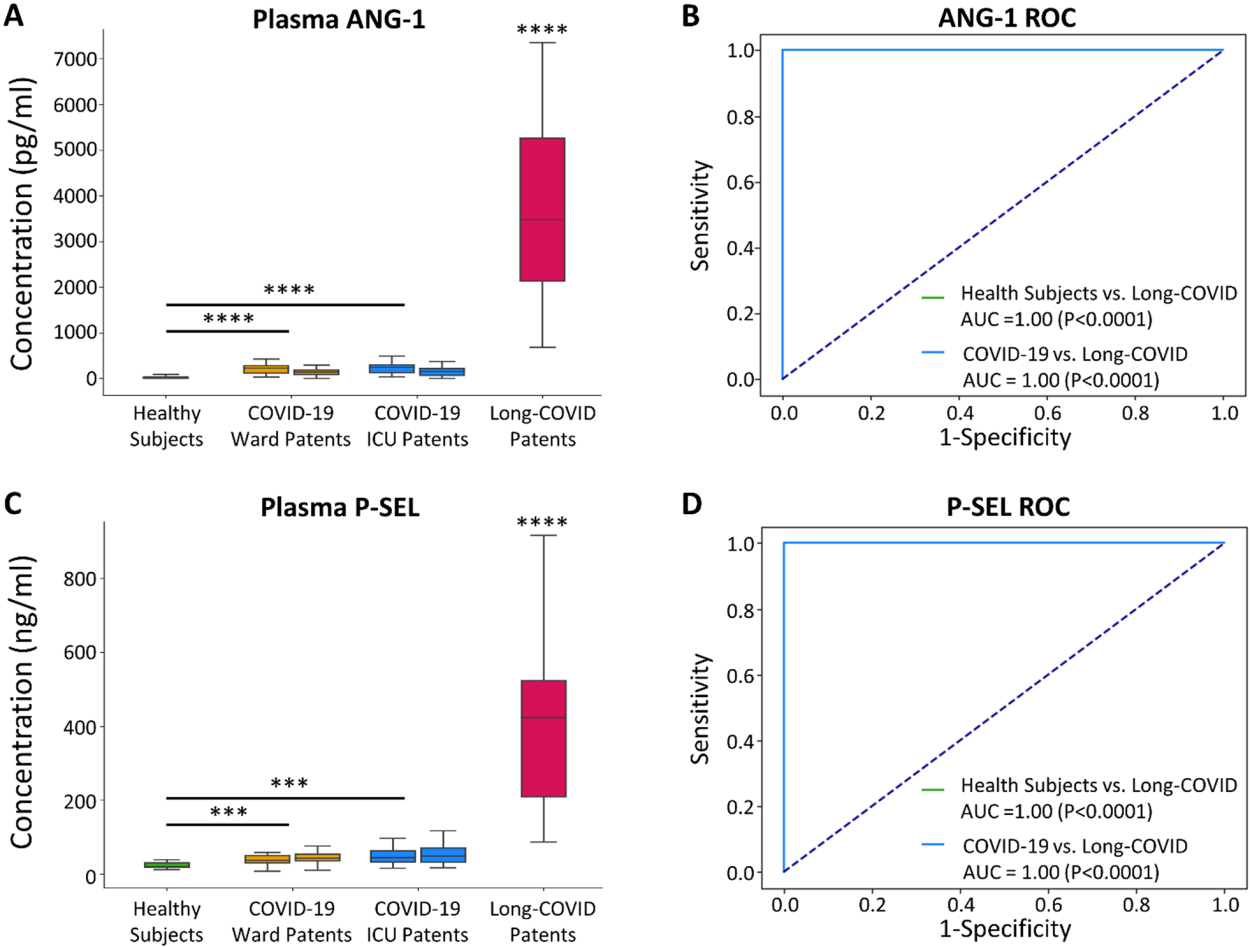
Box plots and receiver operating characteristic (ROC) curves for leading biomarkers, ANG-1 and P-SEL. **A** A boxplot demonstrating significantly elevated blood ANG-1 concentrations in Long-COVID outpatients (****P < 0.0001). **B** ROC curves demonstrating the excellent Long-COVID classification potential of blood ANG-1 versus healthy control subjects (AUC = 1.00, P < 0.0001) and acutely ill COVID-19 inpatients (AUC = 1.00, P < 0.0001). ROC curve for Long-COVID versus healthy control (green) hidden by ROC curve for Long-COVID versus acutely ill COVID-19 patients. **C** A boxplot demonstrating significantly elevated blood P-SEL concentrations in Long-COVID outpatients (****P < 0.0001; ***P < 0.001). **D** ROC curves demonstrating the excellent Long-COVID classification potential of blood P-SEL versus healthy control subjects (AUC = 1.00, P < 0.0001) and acutely ill COVID-19 inpatients (AUC = 1.00, P < 0.0001). ROC curve for Long-COVID versus healthy control (green) hidden by ROC curve for Long-COVID versus acutely ill COVID-19 patients

An individual boxplot comparison of the concentrations of the leading two vascular transformation biomarkers, ANG-1 and P-SEL, illustrated that all were significantly elevated (P < 0.0001) in Long-COVID patients as compared to the other cohorts (Fig. 3A, C). ROC curve analyses demonstrated excellent diagnostic potential for both ANG-1 (AUC 1.0; P < 0.0001; Fig. 3B) and P-SEL (AUCs 1.0; P < 0.0001; Fig. 3D). ROC curve analyses for the remaining fourteen vascular transformation blood biomarkers are listed in Additional file 1: Table S5.

**Fig. 4:**
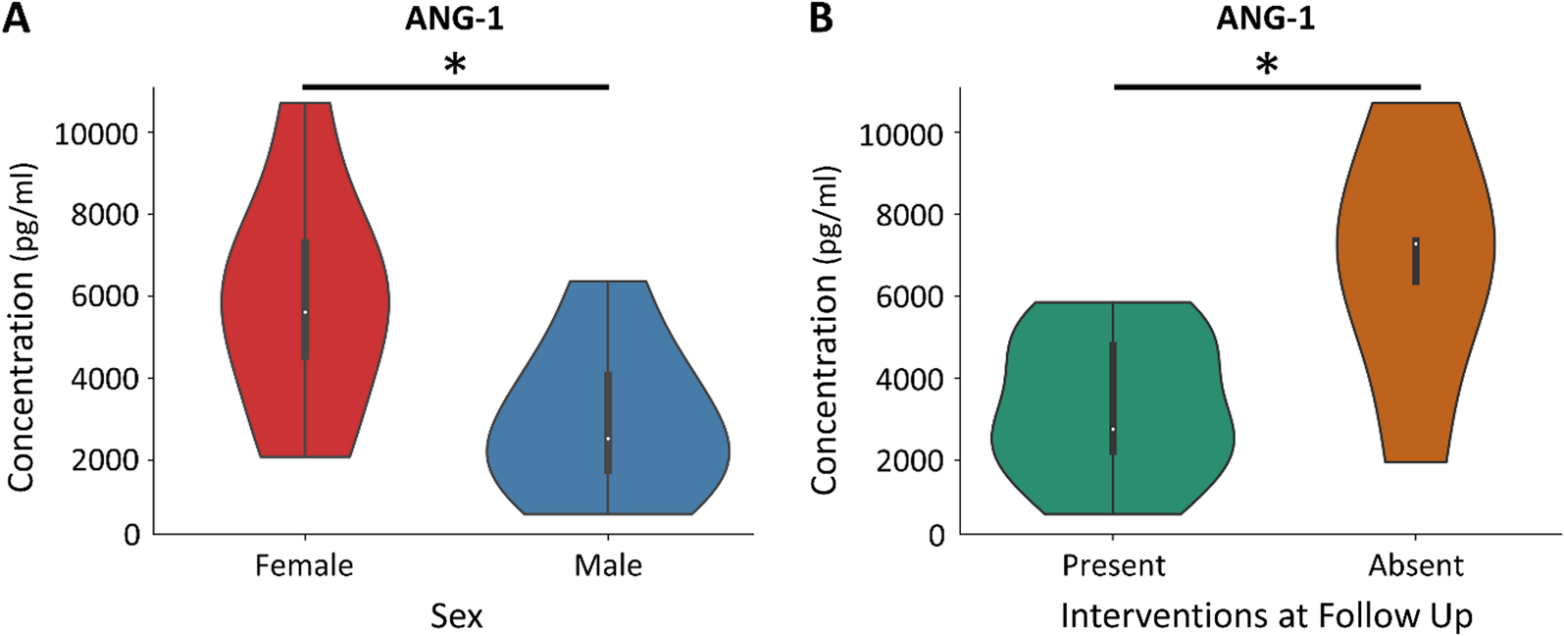
Violin plots demonstrating ANG-1 distribution in Long-COVID patients relative to sex and interventions at follow-up. **A** A violin plot demonstrating significantly elevated concentration of blood ANG-1 in Long-COVID female outpatients (*P < 0.05). **B** A violin plot demonstrating significantly elevated concentration of blood ANG-1 in Long-COVID outpatients that had no interventions at follow-up (*P < 0.05). Given the limited number of patients within this subgroup, the data was not corrected for multiple comparisons and should be considered exploratory

The concentrations of the leading 2 biomarkers, ANG-1 and P-SEL, were compared between the demographics and clinical presentations of the Long-COVID outpatients. Only ANG-1 was significant between females and males (P = 0.028), with females having a higher median ANG-1 blood concentration (Fig. 4A). The higher plasma concentrations of ANG-1 in Long-COVID outpatients was not influenced by sex, as verified by subgroup analysis (data not shown). There were also significant differences in ANG-1 concentrations for Long-COVID outpatients that had an intervention at their follow-up compared to those that had no intervention (P = 0.038). Those without an intervention had a higher median concentration of ANG-1 than those with an intervention at follow-up (Fig. 4B). There was no association between those Long-COVID outpatients that had an intervention on follow-up and the sex of the Long-COVID patients as determined by a Fisher Exact Test (P = 0.69).

The results from the test population were validated in a consecutive, secondary cohort of Long-COVID patients. The baseline demographic characteristics, comorbidities, laboratory measurements, interventions, and chest x-ray findings of Long-COVID outpatients (Additional file 1: Table S6) were similar to the original test population (Table 1). Plasma concentrations of both ANG-1 and P-SEL were significantly elevated in the Long-COVID cohort, when compared to age- and sex-matched healthy control subjects (P = 0.0001; n = 34) and the biomarker concentrations were at or above the previously established cut-off values (Additional file 1: Fig. S1). The biomarker distribution of Long-COVID patients in the validation cohort were clustered at approximately 100 days post acute infection.

## Discussion

In this study, we measured 16 vascular transformation blood biomarkers obtained from age- and sex-matched Long-COVID outpatients, Ward COVID-19 inpatients, ICU COVID-19 inpatients and healthy control subjects. To identify the leading biomarkers distinguishing Long-COVID patients from the other cohorts, we utilized both conventional statistics and state-of-the-art machine learning. Our data indicate a unique Long-COVID blood proteome; two vascular transformation biomarkers were identified that distinguished Long-COVID patients from acutely ill COVID-19 inpatients and healthy control subjects (classification accuracy of 96%). Our data suggest that one or both of the two leading biomarkers, ANG-1 and P-SEL, have potential as disease biomarkers. Moreover, given the primary roles of these two biomarkers in angiogenesis, accelerators or inhibitors of microvascular re-modelling may provide therapeutic potential for Long-COVID patients. The test data was validated in a consecutive, secondary Long-COVID patient cohort.

Our patient cohorts were similar to those reported in earlier studies with regards to demographics, comorbidities and clinical presentation. For example, Long-COVID patients were more likely to be older, have greater body mass index and be female sex (Sudre et al. 2021). They suffered diffuse symptoms, such as fatigue, post-exertional malaise, anosmia and cognitive dysfunction, and across multiple organ systems (Davis et al. 2021; Carfì et al. 2020). With regards to acutely ill COVID-19 patients, they were similar to those reported in earlier cohorts (Myers et al. 2020; Bhatraju 2020; Zhou et al. 2020; Wu et al. 2020), and as per multiple studies, suffered significant inflammatory and thrombotic mechanisms (Fraser et al. 2020a; Fraser et al. 2020c; Fraser et al. 2020d; Gill et al. 2020), associated with microvascular injury (Fraser et al. 2020b).

Our study has identified 14 vascular transformation proteins that are significantly elevated in Long-COVID outpatients, with the leading two proteins (ANG-1 and P-SEL) accurately identifying Long-COVID status. The strength of the leading two proteins for identifying Long-COVID status was demonstrated with both conventional statistics as well as state-of-the-art machine learning techniques. We also provide a rank order listing of the importance of these two molecules relative to the full set of significantly different molecules, resulting in a limited biomarker profile for efficient clinical translatability. The molecules are critical for vascular transformation and suggest they play a wound-healing role in the Long-COVID patients. Information regarding the function of the other molecules is provided in the supplementary documents.

ANG-1, part of the angiopoietin family, is a secreted glycoprotein ligand that induces the tyrosine phosphorylation of TIE2 tyrosine kinase expressed exclusively in vascular endothelial cells and pericytes (Davis et al. 1996). Previous studies have shown ANG-1 to have vasculature protective effects including suppressing plasma leakage, inhibiting vascular inflammation, preventing endothelial death, and enlargement of existing vessels (Brindle et al. 2006; Thurston 2002). The Angiopoietin-TIE2 pathway defends against acute or chronic lung injury and disruptions in the pathway may lead to deterioration in microvascular integrity (Sack et al. 2020). ANG-2, an Angiopoietin-TIE2 antagonist, disrupts the vascular barrier and its elevation is associated with COVID-19 ICU mortality (Vassiliou et al. 2021). As COVID-19 patients have increased angiogenesis due to the endothelial injury (Ackermann 2020), the significantly elevated ANG-1 observed in our Long-COVID patients may represent a long-term, wound-repairing angiogenesis response.

P-SEL is a type-1 transmembrane glycoprotein that is expressed on endothelial cells and platelets (Tvaroška et al. 2020). During infection, the endothelium is activated and P-SEL facilitates platelet aggregation and adhesion (Furie and Furie 2004; Lorant et al. 1993). P-SEL also serves a critical role in angiogenesis by promoting early inflammatory mononuclear cell proliferation (Egami et al. 2006), and mediating endothelial cell migration (Morbidelli et al. 1998). P-SEL is acutely elevated in COVID-19, and it is associated with COVID-19 symptom severity (Fraser et al. 2020e; Venter et al. 2020; Goshua et al. 2020; Yatim, et al. 2021). Lymphocyte binding along specialized high endothelial venules is initiated by P-SEL (Diacovo et al. 1996), and may be associated with our observation of elevated lymphocytes in Long-COVID outpatients, relative to acutely ill COVID-19 inpatients. We could not establish an association between P-SEL and platelet number, suggesting the role of P-SEL in Long-COVID may be skewed towards angiogenesis and lymphocyte migration, rather than platelet aggregation.

Accumulating evidence suggests that females have a greater chance of being affected by Long-COVID (Sudre et al. 2021; Bai 2021). Given the sex- and age-matched nature of our study, the Long-COVID group has roughly an equal number of males (56.5%) and females (43.5%), and thus we cannot corroborate those findings directly. However, ANG-1 was significantly elevated in our female Long-COVID patients, as opposed to males.

Our study has also identified ANG-1 to be significantly elevated in Long-COVID individuals receiving no interventions at follow-up. There were no sex differences for individuals receiving interventions and those without interventions. These latter findings suggest that ANG-1 is critically important for angiogenesis and it is protective at higher levels, perhaps by hastening the healing response.

Long-COVID has been compared to Myalgic encephalomyelitis/chronic fatigue syndrome (ME/CFS), which is an overwhelming fatigue that is not improved by rest and worsened by any physical or mental exertion. Our data suggests that Long-COVID is biochemically distinct from ME/CFS as all 14 vascular transformation biomarkers increased significantly in plasma from Long-COVID patients when compared to healthy control subjects. In contrast, plasma levels of P-SEL, MMP-1, ICAM-1, VEGF-A and VEGF-D either do not change with ME/CFS or are depressed (VanElzakker et al. 2019; Roerink et al. 2017; Roerink et al. 2018). In addition, plasma ANG-1 is unchanged or depressed in common autoimmune and inflammatory diseases, such as rheumatoid arthritis (Senna et al. 2013) and systemic lupus erythematosus (Kümpers et al. 2009).

Our study has identified 14 significantly elevated vascular transformation biomarkers and developed a simple two-protein profile for Long-COVID status; however, our study has several limitations. First, our study investigated a conservative number of patients within each patient group. Nonetheless, we ensured robust analysis via non-parametric statistics and conservative machine learning techniques, and we validated the results in a larger independent cohort. Second, our data show elevated biomarkers in Long-COVID patients at one to six months (median 98.5 days) after acute infection, with clustering at approximately 100 days, but we did not have longitudinal samples from each of these patients to determine individual biomarker resolution. Third, our results demonstrate a potential for one or more vascular transformation biomarkers to identify Long-COVID status; however, we cannot be sure that the same biomarkers may cross-identify with other pathologies. Nonetheless, development of diffuse symptoms and biomarker changes after a PCR-positive acute illness, or in tandem with SARS-CoV-2 nucleocapsid antibody testing, would make cross-reactivity unlikely. Lastly, our comparison of the two selected proteins and clinical/demographic information was exploratory due to limited cohort patient numbers, but given the significant data and scarcity of knowledge on the altered physiological pathways in Long-COVID patients, our findings still provide valuable insights.

## Conclusion

Long-COVID diagnosis and treatment suffers from lack of diagnostic biomarkers and therapeutic targets. In this study, we identify two diagnostic biomarkers (ANG-1 and P-SEL), as well as angiogenesis as a potential therapeutic target. These results are valuable for future hypothesis-generating studies analyzing Long-COVID at a molecular level, developing diagnostic tests, and investigating therapeutic targets. Future studies should also correlate vascular transformation markers with other inflammatory markers to better define potential causal pathways.

## Supplementary Information

**Additional file 1: Table S1**. Function of 16 Vascular Transformation Blood Biomarkers. **Table S2**. Upper and Lower Limit of Quantification (ng/mL) and the Inter- and Intra-assay %CV for 16 Vascular Transformation Blood Biomarkers. **Table S3**: Vascular Transformation Blood Biomarker Concentrations. **Table S4**. Pair-Wise Comparisons of 16 Vascular Transformation Blood Biomarkers. **Table S5**. Classification Accuracy (Random Forest) and Area-Under-the-Curve (ROC Curve Analyses) of 16 Vascular Transformation Blood Biomarkers. **Table S6**. Long-COVID Validation Cohort Outpatient Demographics and Clinical Data. **Fig. S1**. Boxplots and plasma concentrations days after acute infection for ANG-1 and P-SEL in a validation cohort. A) A boxplot demonstrating significantly elevated blood ANG-1 concentrations in Long-COVID outpatients (**** P=0.0001). B) A plot demonstrated ANG-1 concentration versus time after acute infection. A cut-off value adopted from ROC analyses of the test population was calculated. C) A boxplot demonstrating significantly elevated blood P-SEL concentrations in Long-COVID outpatients (**** P=0.0001). D) A plot demonstrated P-SEL concentration versus time after acute infection. A cut-off value adopted from ROC analyses of the test population was calculated.

## Data Availability

The datasets generated and/or analysed during the current study are available from the corresponding author on reasonable request.

## Abbreviations

ANG-1: Angiopoietin-1
MMP-1: Matrix metalloproteinasaes 1
MODS: Multiple organ dysfunction score
P-SEL: P-selectin
PECAM-1: Platelet endothelial cell adhesion molecule 1
PaLM: Pathology and laboratory medicine
FiO_2_: Fractional Inspired Oxygen
PaO_2_: Partial pressure of oxygen
ROC: Receiver operating characteristic curves
SARS-CoV-2: Severe acute respiratory syndrome coronavirus 2
Syn-1: Syndecan-1
TIE2: TEK tyrosine kinase
VCAM-1: Vascular cell adhesion protein 1
VE-Cad: Vascular endothelial cadherin
VEGF-A: Vascular endothelial growth factor A
VEGF-D: Vascular endothelial growth factor d
VEGF-R2: Vascular endothelial growth factor receptor 2
VEGF-R3: Vascular endothelial growth factor receptor 3
VLA-4: Very late antigen-4
t-SNE: T-distributed stochastic nearest neighbour embedding algorithm

## References

[CR1] Ackermann M, et al. Pulmonary vascular endothelialitis, thrombosis, and angiogenesis in Covid-19. N Engl J Med. 2020. 10.1056/NEJMoa2015432 PMC7412750 32437596

[CR2] Bai F, et al. Female gender is associated with long COVID syndrome: a prospective cohort study. Clin Microbiol Infect. 2021. 10.1016/j.cmi.2021.11.002 PMC8575536 34763058

[CR3] Bertsimas D (2020). COVID-19 mortality risk assessment: an international multi-center study. PLoS ONE.

[CR4] Bhatraju PK, et al. COVID-19 in critically ill patients in the seattle region—case series. N Engl J Med. 2020. 10.1056/NEJMoa2004500 PMC7143164 32227758

[CR5] Bradley AP (1997). The use of the area under the ROC curve in the evaluation of machine learning algorithms. Pattern Recogn.

[CR6] Brindle NPJ, Saharinen P, Alitalo K (2006). Signaling and functions of angiopoietin-1 in vascular protection. Circ Res.

[CR7] Brisson AR, Matsui D, Rieder MJ, Fraser DD (2012). Translational research in pediatrics: tissue sampling and biobanking. Pediatrics.

[CR8] Cani E, et al. Immunothrombosis Biomarkers for Distinguishing Coronavirus Disease 2019 Patients From Noncoronavirus Disease Septic Patients With Pneumonia and for Predicting ICU Mortality. Crit Care Explor. 2021;3. 10.1097/CCE.0000000000000588 PMC8718216 34984340

[CR9] Carfì A, Bernabei R, Landi F (2020). Persistent symptoms in patients after acute COVID-19. JAMA.

[CR10] CDC 2019-Novel Coronavirus (2019-nCoV) Real-Time RT-PCR Diagnostic Panel [Internet]. Available from: https://www.fda.gov/media/134922/download. 10.1371/journal.pone.0260487 PMC8673615 34910739

[CR11] Crook H, Raza S, Nowell J, Young M, Edison P. Long covid—mechanisms, risk factors, and management. BMJ. 2021;n1648. 10.1136/bmj.n1648 34312178

[CR12] Davis S (1996). Isolation of angiopoietin-1, a ligand for the TIE2 receptor, by secretion-trap expression cloning. Cell.

[CR13] Davis HE (2021). Characterizing long COVID in an international cohort: 7 months of symptoms and their impact. EClinicalMedicine.

[CR14] Diacovo TG, Puri KD, Warnock RA, Springer TA, Von Andrian UH (1996). Platelet-mediated lymphocyte delivery to high endothelial venules. Science.

[CR15] Egami K, Murohara T, Aoki M, Matsuishi T (2006). Ischemia-induced angiogenesis: role of inflammatory response mediated by P-selectin. J Leukoc Biol.

[CR16] Fraser DD (2020). Inflammation profiling of critically ill coronavirus disease 2019 patients. Crit Care Explor.

[CR17] Fraser DD (2020). Endothelial injury and glycocalyx degradation in critically ill coronavirus disease 2019 patients: implications for microvascular platelet aggregation. Crit Care Explor.

[CR18] Fraser DD (2020). Novel outcome biomarkers identified with targeted proteomic analyses of plasma from critically ill coronavirus disease 2019 patients. Crit Care Explor.

[CR19] Fraser DD (2020). Metabolomics profiling of critically ill coronavirus disease 2019 patients: identification of diagnostic and prognostic biomarkers. Crit Care Explor.

[CR20] Furie B, Furie BC (2004). Role of platelet P-selectin and microparticle PSGL-1 in thrombus formation. Trends Mol Med.

[CR21] Garvin MR, et al. A mechanistic model and therapeutic interventions for COVID-19 involving a RAS-mediated bradykinin storm. Elife. 2020;9. 10.7554/eLife.59177 PMC7410499 32633718

[CR22] Gavriilaki E, Eftychidis I, Papassotiriou I (2021). Update on endothelial dysfunction in COVID-19: severe disease, long COVID-19 and pediatric characteristics. J Lab Med.

[CR23] Gill SE (2020). Transcriptional profiling of leukocytes in critically ill COVID19 patients: implications for interferon response and coagulation. Intensive Care Med Exp.

[CR24] Gillio-Meina C, Cepinskas G, Cecchini EL, Fraser DD (2013). Translational research in pediatrics II: blood collection, processing, shipping, and storage. Pediatrics.

[CR25] Goshua G (2020). Endotheliopathy in COVID-19-associated coagulopathy: evidence from a single-centre, cross-sectional study. Lancet Haematol.

[CR26] Harrison AG, Lin T, Wang P (2020). Mechanisms of SARS-CoV-2 transmission and pathogenesis. Trends Immunol.

[CR27] NIH HRPP. (2009) Policy: guidelines for limits of blood drawn for research purposes in the clinical Center. M95–9 (rev.) June 5.

[CR28] Huang C (2021). 6-month consequences of COVID-19 in patients discharged from hospital: a cohort study. The Lancet.

[CR29] Jambu M, Jambu M (1991). Chapter 10—classification of Individuals-Variables Data Sets. Exploratory and multivariate data analysis.

[CR30] Juneja GK, et al. Biomarkers of coagulation, endothelial function and fibrinolysis in critically-ill patients with COVID-19: a single-centre prospective longitudinal study. J Thromb Haemost. 2021. 10.1111/jth.15327 PMC8250276 33826233

[CR31] Koyama S, Ishii KJ, Coban C, Akira S (2008). Innate immune response to viral infection. Cytokine.

[CR32] Kümpers P (2009). The Tie2 receptor antagonist angiopoietin 2 facilitates vascular inflammation in systemic lupus erythematosus. Ann Rheum Dis.

[CR33] Kursa MB, Rudnicki WR (2010). Feature selection with the Boruta package. J Stat Softw.

[CR34] Lorant DE (1993). Inflammatory roles of P-selectin. J Clin Investig.

[CR35] Van der Maaten L, Hinton G. Visualizing data using t-SNE. J Mach Learn Res. 2008; 9.

[CR36] Matta J (2022). Association of self-reported COVID-19 infection and SARS-CoV-2 serology test results with persistent physical symptoms among french adults during the COVID-19 pandemic. JAMA Intern Med.

[CR37] Morbidelli L, Brogelli L, Granger HJ, Ziche M (1998). Endothelial cell migration is induced by soluble P-selectin. Life Sci.

[CR38] Myers LC, Parodi SM, Escobar GJ, Liu VX (2020). Characteristics of hospitalized adults with COVID-19 in an integrated health care system in California. JAMA.

[CR39] Priestap F, Kao R, Martin CM. External validation of a prognostic model for intensive care unit mortality: a retrospective study using the Ontario Critical Care Information System. Can J Anaesth. 2020. 10.1007/s12630-020-01686-5 PMC7223438 32383124

[CR40] Raveendran AV, Jayadevan R, Sashidharan S (2021). Long COVID: an overview. Diabetes Metab Syndr.

[CR41] Roerink ME (2017). Cytokine signatures in chronic fatigue syndrome patients: a case control study and the effect of anakinra treatment. J Transl Med.

[CR42] Roerink ME (2018). Pitfalls in cytokine measurements—plasma TGF-β1 in chronic fatigue syndrome. Neth J Med.

[CR43] Sack KD, Kellum JA, Parikh SM (2020). The angiopoietin-Tie2 pathway in critical illness. Crit Care Clin.

[CR44] Salamanna F, Maglio M, Landini MP, Fini M (2020). Body localization of ACE-2: on the trail of the keyhole of SARS-CoV-2. Front Med (Lausanne).

[CR45] Senna MK, Machaly SA, Foda M, Eid N (2013). Baseline angiopoietin-2/angiopoietin-1 (Ang2/Ang1) ratio is correlated with the synovial vascularity measured 1 month later in rheumatoid arthritis. Egypt Rheumatol Rehabil.

[CR46] Singer M (2016). The third international consensus definitions for sepsis and septic shock (Sepsis-3). JAMA.

[CR47] Sudre CH (2021). Attributes and predictors of long COVID. Nat Med.

[CR48] Tang C, Garreau D, von Luxburg U. When do random forests fail? In: NeurIPS. 2018; pp. 2987–2997.

[CR49] Thurston G (2002). Complementary actions of VEGF and Angiopoietin-1 on blood vessel growth and leakage*. J Anat.

[CR50] Tvaroška I, Selvaraj C, Koča J (2020). Selectins—the two Dr. Jekyll and Mr. Hyde faces of adhesion molecules—a review. Molecules.

[CR51] VanElzakker MB, Brumfield SA, Lara Mejia PS. Neuroinflammation and Cytokines in Myalgic Encephalomyelitis/Chronic Fatigue Syndrome (ME/CFS): a critical review of research methods. Front Neurol 2019; 9. 10.3389/fneur.2019.00316 PMC6454267 31001197

[CR52] Vassiliou AG (2021). ICU admission levels of endothelial biomarkers as predictors of mortality in critically ill COVID-19 patients. Cells.

[CR53] Venter C (2020). Erythrocyte, platelet, serum ferritin, and P-selectin pathophysiology implicated in severe hypercoagulation and vascular complications in COVID-19. Int J Mol Sci.

[CR54] Wu C (2020). Risk factors associated with acute respiratory distress syndrome and death in patients with coronavirus disease 2019 pneumonia in Wuhan, China. JAMA Intern Med.

[CR55] Yatim N, et al. Platelet activation in critically ill COVID-19 patients. Annal Intensive Care 2021; 11. 10.1186/s13613-021-00899-1 PMC8286043 34273008

[CR56] Yuki K, Fujiogi M, Koutsogiannaki S (2020). COVID-19 pathophysiology: a review. Clin Immunol.

[CR57] Zhou F (2020). Clinical course and risk factors for mortality of adult inpatients with COVID-19 in Wuhan, China: a retrospective cohort study. Lancet.

