# Supplementary material for: Elevated vascular transformation blood biomarkers in Long-COVID indicate angiogenesis as a key pathophysiological mechanism
Source: Mol Med. 2022 Oct 10;28:122. doi: 10.1186/s10020-022-00548-8 (PMC9549814; doi:10.1186/s10020-022-00548-8)
Supplement: Supplementary file 1 — Additional file 1: Table S1. Function of 16 Vascular Transformation Blood Biomarkers. Table S2. Upper and Lower Limit of Quantification (ng/mL) and the Inter- and Intra-assay %CV for 16 Vascular Transformation Blood Biomarkers. Table S3: Vascular Transformation Blood Biomarker Concentrations. Table S4. Pair-Wise Comparisons of 16 Vascular Transformation Blood Biomarkers. Table S5. Classification Accuracy (Random Forest) and Area-Under-the-Curve (ROC Curve Analyses) of 16 Vascular Transformation Blood Biomarkers. Table S6. Long-COVID Validation Cohort Outpatient Demographics and Clinical Data. Fig. S1. Boxplots and plasma concentrations days after acute infection for ANG-1 and P-SEL in a validation cohort. A) A boxplot demonstrating significantly elevated blood ANG-1 concentrations in Long-COVID outpatients (**** P=0.0001). B) A plot demonstrated ANG-1 concentration versus time after acute infection. A cut-off value adopted from ROC analyses of the test population was calculated. C) A boxplot demonstrating significantly elevated blood P-SEL concentrations in Long-COVID outpatients (**** P=0.0001). D) A plot demonstrated P-SEL concentration versus time after acute infection. A cut-off value adopted from ROC analyses of the test population was calculated. [file 10020_2022_548_MOESM1_ESM.docx]

**Supplemental Table 1. Function of 16 Vascular Transformation Blood Biomarkers.**

|  | **Biomarker** | **Function** |
| --- | --- | --- |
| 1 | ANG-1 | Angiopoietin-1 (ANG-1), part of the angiopoietin family, is expressed in vascular smooth muscle cells, induces TIE2 tyrosine kinase and activates angiogenesis and vascular protective effects including suppressing plasma leakage, inhibiting vascular inflammation, preventing endothelial death, and enlargement of existing vessels. |
| 2 | P-SEL | P-Selectin (P-SEL), expressed on endothelial cells and platelets, facilitates platelet aggregation, platelet adhesion, endothelial cell migration and mononuclear cell proliferation. |
| 3 | MMP-1 | Matrix Metalloproteinases 1 (MMP-1) cleaves peptide bonds of the extracellular matrix during the initial angiogenesis and wound healing stages. Over, unregulated expression can lead to harmful processes including tumor-promoting proliferation, invasion, and metastasis. |
| 4 | VE-Cad | Vascular Endothelial Cadherin (VE-Cad) is a cell-cell adhesion glycoprotein and critical to maintaining a restrictive endothelial barrier. Blocking VE-Cadherin inhibits angiogenesis and the formation of vascular structures in vitro. |
| 5 | Syn-1 | Syndecan-1 (Syn-1) is expressed primarily on epithelial cells but also endothelial cells and leukocytes. Syn-1 binds ligands, including ICAM-1 and VCAM-1, via its Heparan sulfate chain. Syn-1 in inflammation negatively regulates leukocyte adhesion and migration. |
| 6 | Endoglin | Auxiliary receptor for the transforming growth factor-ß (TGF- ß). Elevated expression during inflammation and wound healing, is critical for angiogenesis and maintaining normal vascular architecture. |
| 7 | PECAM-1 | Platelet endothelial cell adhesion molecule-1 (PECAM-1) is a cell-cell adhesion molecule on vascular cells including platelets, endothelial cells, lymphocytes, and is involved in leukocyte transmigration, signaling, angiogenesis. |
| 8 | VEGF-A | Vascular endothelial growth factor A (VEGF-A) generated by hypoxia-increased VEGF gene transcription, resulting in angiogenesis processes: cell migration, proliferation, microvascular permeability, and MMP activity. |
| 9 | ICAM-1 | Increased Intercellular adhesion molecule-1 (ICAM-1) expression in endothelial cells, macrophages, and lymphocytes by proinflammatory cytokines (IL-1, TNF). Facilitates leukocyte LFA-1 binding to endothelial cells and enables leukocyte transmigration. |
| 10 | VLA-4 | Very Late Antigen-4 (Integrin α4ß1, VLA-4), part of integrins family of immune cell adhesion receptors where VLA-4 specifically facilitates leukocyte arrest on endothelium and transmigration during inflammation. Expression regulated by growth factors or chemokines, common ligand is VCAM-4 and fibronectin. |
| 11 | E-SEL | E-Selectin (E-SEL) is expressed by endothelial cells when upregulated by inflammatory cytokines (TNF-α and IL-1ß) and binds carbohydrate ligands expressed by leukocytes to facilitate recruitment. |
| 12 | Thrombomodulin | A transmembrane glycoprotein expressed on endothelial, immune, lung alveolar epithelial cells. Decreases intravascular coagulation by 1. Binding and sequestering thrombin (converts fibrinogen and activates platelets) 2. Thrombin-thrombomodulin pathway downregulates thrombin generation. |
| 13 | VEGF-R2 | Vascular endothelial growth factor receptor-2 (VEGF-R2) in vascular endothelial cells facilitates VEGF-A, -C, and -D binding and signals for angiogenesis and vasculogenic activation. VEGF-R2 also regulates endothelial cell growth, differentiation, and migration. |
| 14 | VEGF-R3 | Vascular endothelial growth factor receptor-3 (VEGF-R3) primarily in lymphatic endothelial cells binds VEGF-C and -D and signals for lymphangiogenesis. VEGF-R3 is also implicated in regulating angiogenesis and vascular network formation via VEGF-A. |
| 15 | VCAM-1 | Vascular cell adhesion protein 1 (VCAM-1) is part of the immunoglobulin family, expressed in endothelial cells and functions as a ligand for VLA-4, and regulates leukocyte transmigration. |
| 16 | VEGF-D | Vascular endothelial growth factor D (VEGF-D), expressed in all tissues but predominantly in lung and skin, binds VEGF-R2 and VEGF-R3 to increase angiogenesis and lymphangiogenesis. Unprocessed VEGF-D has a poor affinity to VEGF-R2, proteolytic processed VEGF-D increases affinity to VEGF-R2 and boosts the affinity of VEGF-R3. |

**Supplemental Table 2: Upper and Lower Limit of Quantification (ng/mL) and the Inter- and Intra-assay %CV for the 16 Vascular Transformation Blood Biomarkers.**

| **Biomarker^1^** | **ULOQ/LLOQ (ng/ml)** | **Inter-assay %CV** | **Intra-assay %CV** |
| --- | --- | --- | --- |
| ANG-1 | 58.375 / 0.0570 | <10% | <10% |
| E-SEL | 1849.5 / 1.8062 | <10% | <10% |
| MMP-1 | 46.1 / 0.0113 | <10% | <10% |
| PECAM-1 | 1118.8 / 1.0926 | <15% | <15% |
| P-SEL | 5462.2 / 1.3335 | <10% | <10% |
| Syn-1 | 45.2 / 0.0110 | <10% | <10% |
| Thrombomodulin | 93.4 / 0.0228 | <10% | <10% |
| VEGF-A | 46.8 / 0.0114 | <5% | <10% |
| VEGF-D | 11.9 / 0.0029 | <5% | <5% |
| VEGF-R2 | 330.0 / 0.3223 | <5% | <10% |
| VEGF-R3 | 3653.0 / 3.5674 | <10% | <10% |
| VLA-4 | 420.7 / 0.1027 | <5% | <10% |
|  |  |  |  |
| **Biomarker^2^** | **ULOQ/LLOQ (ng/ml)** | **Inter-assay %CV** | **Intra-assay %CV** |
| Endoglin | 2.3 / 0.0005 | <10% | <10% |
| ICAM-1 | 493.2 / 0.1204 | <10% | <10% |
| VCAM-1 | 38.9 / 0.0095 | <10% | <10% |
| VE-Cad | 50.0 / 0.0122 | <5% | <5% |

^1^ Endothelial Injury Marker 12plex Human ProcartaPlex™ Panel,

EPX120-15849-901. ^2^ Mix&Match 4plex ProcartaPlex™ Panel

ULOQ/LLOQ were determined in quality control of the ProcartaPlex kits for standard points achieving 80-120% of back calculated observed versus expected levels on 5pl fit curve, giving the valid assay range. Inter- and Intra-assay CV levels were calculated from 6 samples measured in 6 replicates on 3 plates during assay-validation. For evaluation of intra-assay %CV, the %CV of the 6 replicates is calculated. For evaluation of inter-assay %CV, the variance of the obtained sample values (mean of 6 replicates) on plate-1, plate-2, and plate-3 is calculated.

**Supplemental Table 3: Vascular Transformation Blood Biomarker Concentrations.**

| Biomarker | Healthy  Subjects | COVID-19  Ward | COVID-19 ICU | Long-  COVID |
| --- | --- | --- | --- | --- |
| ANG-1 | 0.0  (0.0-  36.0) | 182.6  (101.4-  246.6) | 180.0 (120.3-283.7) | 3474.9 (2130.8-5259.1) |
| P-SEL | 20.6  (18.0-  30.4) | 39.3  (34.7-  51.3) | 46.5  (32.3-  64.9) | 423.5  (209.0  -524.5) |
| MMP-1 | 31.4  (17.4-  48.5) | 64.4  (50.8-  95.0) | 113.5  (69.1-  140.3) | 1051.0 (461.3-516.6) |
| VE-Cad | 1070  (881-  1346) | 708  (615-  895) | 708  (582-  964) | 1739  (1620-  2529) |
| Syn-1 | 0.4  (0.3-  0.6) | 0.7  (0.5-  0.9) | 1.1  (0.8-  1.7) | 0.7  (0.6-  0.9) |
| Endoglin | 3.3  (2.8-  10.0) | 2.2  (1.5-  4.3) | 2.2  (1.2-  4.3) | 8.1  (5.7-  19.8) |
| PECAM-1 | 22.0  (20.2-  26.3) | 19.2  (14.4-  24.6) | 20.8  (13.7-  31.9) | 45.4  (34.7-  54.5) |
| VEGF-A | 0.2  (0.1-  0.3) | 0.3  (0.2-  0.5) | 0.5  (0.4-  1.1) | 1.5  (1.0-  2.7) |
| ICAM-1 | 288  (238-  791) | 518  (352-  1234) | 688  (407-  1228) | 937  (835-  2114) |
| VLA-4 | 0.1  (0.0-  0.1) | 0.1  (0.0-  0.1) | 0.1  (0.0-  0.2) | 0.3  (0.2-  0.4) |
| E-SEL | 34.3  (28.9-  43.6) | 25.6  (19.1-  32.4) | 39.1  (28.6-  50.0) | 67.4  (52.4-  81.2) |
| Thrombomodulin | 0.5  (0.3-  0.6) | 0.4  (0.2-  0.6) | 0.6  (0.5-  0.9) | 1.4  (0.6-  2.3) |
| VEGF-R2 | 10.3  (7.7-  11.8) | 11.6  (8.1-  16.0) | 12.2  (9.3-  18.4) | 22.2  (16.7-  26.1) |
| VEGF-R3 | 16.9  (11.1-  21.4) | 17.6  (11.2-  24.0) | 18.5  (10.4-  24.9) | 31.8  (25.3-  40.8) |
| VCAM-1 | 303  (255-  884) | 361  (285-  989) | 541  (339-  1180) | 450  (374-  1165) |
| VEGF-D | 22.2  (12.7-  37.1) | 39.7  (23.3-  57.1) | 34.5  (22.3-  65.4) | 27.3  (12.7-  35.8) |

Median (IQRs) ng/ml, except for ANG-1, MMP-1, and VEGF-D at pg/ml

**Supplemental Table 4. Pair-Wise Comparisons of 16 Vascular Transformation Blood Biomarkers.**

| **Biomarker** | **L-C vs. Healthy** | **L-C vs. Ward** | **L-C vs. ICU** | **Healthy vs. ICU** | **Healthy vs Ward** | | **Ward vs. ICU** |
| --- | --- | --- | --- | --- | --- | --- | --- |
| ANG-1 | <0.0001 | <0.0001 | <0.0001 | <0.0001 | <0.0001 | 1 | |
| P-SEL | <0.0001 | <0.0001 | <0.0001 | 0.0001 | 0.0004 | | 1 |
| MMP-1 | <0.0001 | <0.0001 | <0.0001 | <0.0001 | 0.0005 | | 0.0437 |
| VE-Cad | <0.0001 | <0.0001 | <0.0001 | 0.0014 | 0.0024 | | 1 |
| Syn-1 | 0.0013 | 1 | 0.1485 | <0.0001 | 0.0003 | | 0.0002 |
| Endoglin | 0.0047 | <0.0001 | <0.0001 | 0.0138 | 0.0112 | | 1 |
| PECAM-1 | <0.0001 | <0.0001 | <0.0001 | 1 | 0.4165 | | 0.7330 |
| VEGF-A | <0.0001 | <0.0001 | 0.0015 | <0.0001 | 0.0044 | | 0.0034 |
| ICAM-1 | 0.0016 | 0.0600 | 0.0347 | 0.0048 | 0.1105 | | 1 |
| VLA-4 | <0.0001 | <0.0001 | <0.0001 | 0.8992 | 1 | | 1 |
| E-SEL | <0.0001 | <0.0001 | <0.0001 | 1 | 0.0037 | | <0.0001 |
| Thrombomodulin | 0.0005 | <0.0001 | 0.0156 | 0.1742 | 1 | | 0.0196 |
| VEGF-R2 | <0.0001 | 0.0002 | 0.002 | 0.2953 | 1 | | 1 |
| VEGF-R3 | 0.0006 | 0.0004 | <0.0001 | 1 | 1 | | 1 |
| VCAM-1 | 0.0683 | 0.3346 | 1 | 0.1580 | 1 | | 0.2305 |
| VEGF-D | 1 | 0.1037 | 0.5067 | 0.5073 | 0.0890 | | 1 |

L-C, Long-COVID outpatients; Healthy, health control subjects; Ward, acutely ill COVID-19 ward inpatients; ICU, acutely ill COVID-19 ICU inpatients. Mann-Whitney U test with Bonferroni multiple comparisons correction (n=23/group).

**Supplemental Table 5. Classification Accuracy (Random Forest) and Area-Under-the-Curve (ROC Curve Analyses) of 16 Vascular Transformation Blood Biomarkers.**

|  | L-C vs. Healthy | | L-C vs. COVID-19 | |
| --- | --- | --- | --- | --- |
| Biomarker | Classification Accuracy (%) | AUC | Classification Accuracy (%) | AUC |
| ANG-1 | 98 | 1.00 | 97 | 1.00 |
| P-SEL | 98 | 1.00 | 94 | 1.00 |
| MMP-1 | 97 | 1.00 | 91 | 0.94 |
| VE-Cad | 78 | 0.91 | 84 | 0.96 |
| Syn-1 | 69 | 0.79 | 76 | 0.47 |
| Endoglin | 74 | 0.79 | 78 | 0.88 |
| PECAM-1 | 85 | 0.98 | 86 | 0.96 |
| VEGF-A | 87 | 0.96 | 81 | 0.83 |
| ICAM-1 | 87 | 0.81 | 78 | 0.71 |
| VLA-4 | 70 | 0.86 | 85 | 0.85 |
| E-SEL | 76 | 0.91 | 86 | 0.89 |
| Thrombomodulin | 78 | 0.84 | 80 | 0.82 |
| VEGF-R2 | 82 | 0.92 | 76 | 0.77 |
| VEGF-R3 | 74 | 0.81 | 79 | 0.79 |
| VCAM-1 | 56 | 0.74 | 81 | 0.56 |
| VEGF-D | 41 | 0.52 | 75 | 0.35 |

L-C, Long-COVID outpatients (n=23); Healthy, health control subjects (n=23); COVID-19, Ward and ICU patients combined (n=46).

**Supplemental Table 6. Long-COVID Validation Cohort Outpatient Demographics and Clinical Data.**

| **Initial Infection Variable** | **Patients (n=34)** | |
| --- | --- | --- |
| Age (yrs), median (IQR) | 48.0 (21.8) | |
| Male sex, No. (%) | 13 (38.2) | |
| Diagnostic test: PCR, serology, no. (%) | 34 (100.0) | |
| Vaccination status at infection, no. (%) | 0 (0%) | |
| **Hospitalization, no. (%)** |  | |
| Ward | 12 (35.3) | |
| ICU | 1 (2.9) | |
| **Comorbidities, no. (%)** |  | |
| Diabetes | 4 (11.8) | |
| Hypertension | 6 (17.6) | |
| Coronary artery/heart disease | 4 (11.8) | |
| Chronic/congestive heart failure | 3 (8.8) | |
| Cancer | 2 (5.9) | |
| COPD | 1 (2.9) | |
| Asthma | 6 (17.6) | |
| **Presenting symptoms at infection, no. (%)** |  | |
| Fever | 27 (79.4) | |
| Cough | 28 (82.4) | |
| Anosmia/Ageusia | 16 (47.1) | |
| Pharyngitis | 10 (29.4) | |
| Headache | 20 (58.8) | |
| Confusion/Memory | 6 (17.6) | |
| Myalgias | 20 (58.8) | |
| Dyspnea | 26 (76.5) | |
| Chest pain | 11 (32.4) | |
| Nausea/Vomiting/Diarrhea | 17 (50.0) | |
| **Interventions at infection, no. (%)** |  | |
| Steroids | 11 (33.3) | |
| Remdesivir | 3 (9.1) | |
| Tocilizumab | 2 (6.1) | |
|  |  | |
| **Long-COVID Clinic Variables** | |  |
| Follow up, days from infection onset, median (IQR) | 101.0 (39.2) | |
| **Lingering symptoms at follow up, no. (%)** |  | |
| Respiratory | 25 (73.5) | |
| Cardiovascular | 1 (2.9) | |
| Neurology | 18 (52.9) | |
| Musculoskeletal | 3 (8.8) | |
| Gastro-Intestinal | 1 (2.9) | |
| Balance | 2 (5.9) | |
| Chest pain | 1 (2.9) | |
| Dyspnea | 25 (73.5) | |
| Fatigue | 6 (17.6) | |
| Headache | 3 (8.8) | |
| Low mood | 1 (2.9) | |
| Anxiety | 1 (2.9) | |
| Memory | 7 (20.6) | |
| Paresthesia | 1 (2.9) | |
| Smell/taste | 6 (17.6) | |
| Fever | 1 (2.9) | |
| Confusion | 1 (2.9) | |
| Brain Fog | 2 (5.9) | |
| Myalgias | 2 (5.9) | |
| Neuropathy | 2 (5.9) | |
| Nausea/Vomiting/Diarrhea | 1 (2.9) | |
| Word finding | 1 (2.9) | |
| Sleep | 5 (14.7) | |
| **Laboratories at follow up, median (IQR)** |  | |
| White blood cell count | 7.0 (3.0) | |
| Neutrophils | 4.1 (1.8) | |
| Lymphocytes | 1.9 (0.7) | |
| Hemoglobin | 136.0 (17.0) | |
| Platelets | 279.0 (68.0) | |
| C-Reactive Protein (CRP) | 2.6 (3.8) | |
| Ferritin | 92.8 (130.3) | |
| Lactate Dehydrogenase (LDH) | 193.5 (32.2) | |
| Alanine Aminotransferase (ALT) | 20.0 (13.0) | |
| **Interventions, no. (%)** |  | |
| Symbicort | 15 (44.1) | |
| Ventolin | 1 (2.9) | |
| Lasix | 1 (2.9) | |
| Spiriva | 1 (2.9) | |
| Fluticasone Puffer | 1 (2.9) | |
| Psychology | 1 (2.9) | |
| Physiotherapy | 1 (2.9) | |
| None | 17 (50.0) | |

**Supplemental Figure Legend.**

**Supplemental Figure 1. Box plots and plasma concentrations days after acute infection for ANG-1 and P-SEL in validation cohort. A)** A boxplot demonstrating significantly elevated blood ANG-1 concentrations in Long-COVID outpatients (**** P<0.0001). **B)** A plot demonstrated ANG-1 concentration versus time after acute infection. A cut-off value adopted from ROC analyses of the test population was calculated. **C)** A boxplot demonstrating significantly elevated blood P-SEL concentrations in Long-COVID outpatients (**** P<0.0001). **D)** A plot demonstrated P-SEL concentration versus time after acute infection. A cut-off value adopted from ROC analyses of the test population was calculated.

**Supplemental Figure 1.
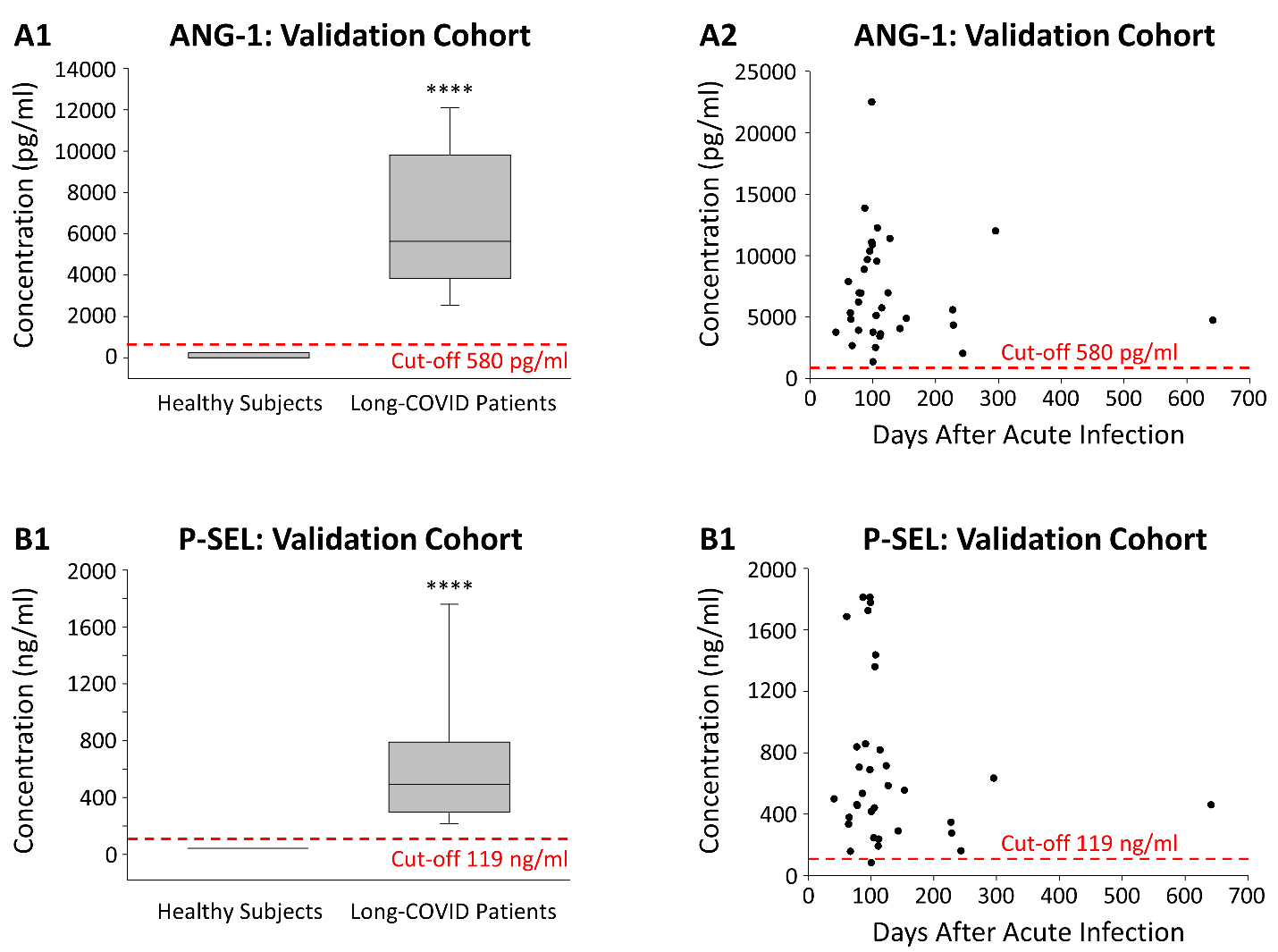
**
